# Identifying the causes of recurrent pregnancy loss in consanguineous couples using whole exome sequencing on the products of miscarriage with no chromosomal abnormalities

**DOI:** 10.1038/s41598-021-86309-9

**Published:** 2021-03-26

**Authors:** Kimia Najafi, Zohreh Mehrjoo, Fariba Ardalani, Siavash Ghaderi-Sohi, Ariana Kariminejad, Roxana Kariminejad, Hossein Najmabadi

**Affiliations:** 1 Genetic Research Center, National Reference Laboratory for Prenatal Diagnosis, University of Social Welfare and Rehabilitation Sciences, Koodakyar Avenue, Daneshjoo Blvd, Evin, Tehran, 1985713834 Iran; 2 Kariminejad‐Najmabadi Pathology and Genetics Center, #2, West Side of Sanat Sq.-Metro Station, Shahrak Gharb, Tehran, 1466713713 Iran

**Keywords:** Genetics, Consanguinity, Sequencing

## Abstract

Recurrent miscarriages occur in about 5% of couples trying to conceive. In the past decade, the products of miscarriage have been studied using array comparative genomic hybridization (a-CGH). Within the last decade, an association has been proposed between miscarriages and single or multigenic changes, introducing the possibility of detecting other underlying genetic factors by whole exome sequencing (WES). We performed a-CGH on the products of miscarriage from 1625 Iranian women in consanguineous or non-consanguineous marriages. WES was carried out on DNA extracted from the products of miscarriage from 20 Iranian women in consanguineous marriages and with earlier normal genetic testing. Using a-CGH, a statistically significant difference was detected between the frequency of imbalances in related vs. unrelated couples (*P* < 0.001). WES positively identified relevant alterations in 11 genes in 65% of cases. In 45% of cases, we were able to classify these variants as pathogenic or likely pathogenic, according to the American College of Medical Genetics and Genomics guidelines, while in the remainder, the variants were classified as of unknown significance. To the best of our knowledge, our study is the first to employ WES on the products of miscarriage in consanguineous families with recurrent miscarriages regardless of the presence of fetal abnormalities. We propose that WES can be helpful in making a diagnosis of lethal disorders in consanguineous couples after prior genetic testing.

## Introduction

According to the American Society for Reproductive Medicine (ASRM) report, recurrent pregnancy loss is defined as two or more miscarriages^1^ and occurs in about 5% of couples trying to conceive^2^. Both maternal and fetal causes can lead to pregnancy loss^3^. Numerical chromosome abnormalities are the most common cause of miscarriage, particularly under 13 weeks^4,5^. However, for the 40–50% of miscarriages with a normal karyotype (euploid miscarriage), the etiology is unknown and the genetic etiology is uncertain^2^. Karyotyping of products of miscarriage as a routine test has its limitations including culture failure rates (10–40%)^6–8^, and maternal cell contamination^7,9,10^, and resolution (usually below 5–10 Mb) is unable to detect minor genomic changes. In the past decade, the products of miscarriage have been studied using chromosomal microarray (CMA) technology (array comparative genomic hybridization (a-CGH))^5,8,11–13^ and clinically relevant copy number variants (CNVs) have been reported in about 1.6–1.8% of miscarriages. CMA overcomes most issues concerning the quality or quantity of fetal samples that have proved problematic in routine chromosomal study. CMA has its limitations including its inability to detect polyploidy, low-grade mosaicism and balanced rearrangements^7^.

In 2013, Larsen et al. proposed an association between miscarriage and single or multigenic changes^14^, introducing the possibility of being able to detect other underlying genetic factors by exome sequencing. Today, next-generation sequencing (NGS) is a crucial tool for pathogenic variants discovery in research and diagnostic settings^15,16^. Exome sequencing has a diagnostic yield of about 25–40% in patients with suspected Mendelian diseases in western populations^17–20^. Carss et al. performed exome sequencing on 30 non-aneuploid fetuses and neonates with diverse structural abnormalities detected by prenatal ultrasound. They identified candidate pathogenic variants and concluded that exome sequencing may substantially increase the detection rate of underlying etiologies of prenatal abnormalities. In 3 out of 30 fetuses, they found highly likely causative variants^15^. In 2018, Mengu Fu et al. performed exome sequencing on 19 products of miscarriage of unrelated couples and reported 36 rare variants associated with miscarriage^21^.

Studies of this kind have led to the use of NGS in prenatal diagnosis for the detection of pathogenic and causal genetic variants below the resolution of CMA^15,22^. The application of NGS in identifying the causes of lethal or abnormal prenatal development, including miscarriages, has been reported since 2012. An estimate of 30% of all mammalian genes are vital for life. Dickinson et al. (2016) identified 410 lethal genes in mice. In general, the diagnosis of lethal genes is challenging for the following reasons: the difficulties of phenotype–genotype correlation, the many potential genes, and the variable phenotypes associated with the same genetic causes^23^. There is controversy as to whether there is any correlation between recurrent pregnancy loss (RPL) and consanguinity. In studies conducted in the Middle East, where consanguineous marriage is culturally prevalent, some showed that the prevalence of RPL is higher in consanguineous couples. Kuntla et al. (2013) concluded that the occurrence of spontaneous miscarriages is higher among women in related vs. unrelated couples in India. In 2010, Rad conducted a study on recurrent miscarriage in Iran and concluded that the prevalence of RPL was higher in the consanguineous group^24^. On the other hand, in 2002, Saad and Jauniaux reported no association between consanguinity and recurrent miscarriage in Qatar and concluded that this finding could be because autosomal recessive alleles are uncommon in the Qatari population or because of the absence of any relationship between consanguinity and recurrent miscarriage^25^. In 2011, Gowri et al. reported that consanguinity does not appear to play a significant role in the etiology of recurrent miscarriage and is not related to recurrent miscarriage based on a study conducted in Oman^26^.

Since 2010, about 1625 cases with pregnancy loss (recurrent or single) have been studied in our center using quantitative fluorescent polymerase chain reaction (QF-PCR) and a-CGH, in which about 20% of clinically relevant CNVs and aneuploidies were detected. About 35% of the cases were from consanguineous couples. The inbreeding coefficients in consanguineous couples were between *F* = 0.125 and *F* = 0.0156. We realized that the detection rate in unrelated couples was higher. The difference between the frequency of imbalances in related vs. unrelated couples was significant (χ2 = 11.4926, *P* < 0.001)^27^. We proposed that it is plausible through the same mechanism by which single gene disorders have a higher prevalence of manifesting disease in consanguineous couples, they can cause lethal genetic disorders leading to pregnancy loss. As miscarriage is etiologically heterogeneous, the selection criteria for the evaluation of this postulate are very important. Whole exome sequencing (WES) on cases that present with a strong Mendelian inheritance pattern is more likely to be successful^28^. Here we have used WES to detect the causes of miscarriage in consanguineous Iranian families with recurrent miscarriages, in whom oligoarray CGH was normal and the maternal causes of miscarriage had been ruled out.

## Materials and methods

Signed informed consent was obtained from couples for participation in this study and publication of data. The research was performed under the National Institute for Medical Research Development, Tehran, Iran (IR.NIMAD.REC.1396.355).

### Subjects

Twenty consanguineous Iranian couples were selected from a pool of 1625 couples in whom oligoarray CGH was normal and maternal causes of miscarriage had been ruled out by Kariminejad–Najmabadi Pathology & Genetics Center. The couples had a history of two or more pregnancy losses (RPL) and the results of previous a-CGH and QF-PCR were normal. Five of the cases had fetal autopsy reports.

The cases were selected from consanguineous couples with spontaneous loss of a pregnancy at less than 20 weeks of gestation and whose fetuses had sufficient quantity of DNA with appropriate quality. To minimize the effect of maternal causes of miscarriage, we chose families who had a history of one normal child, or one pregnancy beyond the second trimester or whose fetus showed evidence of fetal abnormalities (non-immune hydrops fetalis (NIHF), cardiac anomalies, etc.)^29^. None of the cases had induced abortion or prior abnormality detected in ultrasound. (Table 1).

**Table 1:** Families obstetrics history.

Cases ID	Prior abortions	Born child	Pregnancy over second trimester	Anomalies in prior pregnancies
82169	2	1	–	Hydrops fetalis
84904	3	–	1	No fetal heart rate
88254	2	1	–	–
88588	2	1	–	–
89506	3	1	–	–
89943	4	1	1	–
90179	2	–	1	–
90202	3	1	–	–
90377	5	–	2	–
90759	2	–	1	Hydrocephaly
91414	2	1	–	Hydrocephaly
91926	2	–	1	Kidney dysplasia
92386	2	–	1	Hydrocephaly and absent nasal bone
93272	4	–	1	No fetal heart rate
93926	2	–	1	Bone dysplasia
94162	2	–	1	–
94947	3	–	1	–
95136	4	1	2	–
76452	2	–	1	–
97406	2	–	1	–

All experimental protocols were approved by National Institute for Medical Research Development committee.

### Whole exome sequencing

We used archival genomic DNA from the products of spontaneous miscarriage and extracted DNA from the peripheral blood of parents. All DNA was extracted using the conventional salting out method^30^. For fetuses or the products of miscarriage, DNA was extracted from chronic villi of the products of miscarriage and where fetal tissue was available, from the quadriceps muscle. Where possible, we saved some fetal tissue in a freezer at –80 °C. We checked the quality and quantity of DNA using a Nanodrop spectrophotometer (NanoDrop Technologies, Inc., Wilmington, DE, USA) and gel electrophoresis. Samples were enriched using the Agilent SureSelectXT Human All Exon V6 platform (Agilent Technologies Inc, Santa Clara, CA, USA). Whole exome sequencing was performed using an Illumina HiSeq2000 platform (Illumina Inc., San Diego, CA, USA)^31^. All methods were carried out in accordance with ACMG guidelines and regulations.

### Data analysis

Sequence reads were aligned to human GRCH37 using the Burrows–Wheeler Aligner (BWA) with the MEM algorithm^32^. Data processing and variant (SNPs and indels) discovery were performed according to the Genome Analysis Toolkit (GATK) best practices workflow^33,34^. Variant annotation was performed using ANNOVAR software^35^. To refine the list of causative variants, additional filtering was applied as follows. We kept exonic, splicing, UTR3 and UTR5 regions with the following criteria:

1. Rarely seen in populations (cut of 0.01) based on the Genome Aggregation Consortium (http://gnomad.broadinstitute.org/), 1000 genome project (http://www.internationalgenome.org/data), The Exome Aggregation Consortium (http://exac.broadinstitute.org/), NHLBI Exome Sequencing Project (http://evs.gs.washington.edu/EVS/) and Iranome databases (http://www.iranome.ir/)^36–39^.

2. Predicted to have functional consequences based on prediction scores such as SIFT (http://sift.bii.a-star.edu.sg/), PolyPhen2 (http://genetics.bwh.harvard.edu/pph2/), MutationTaster (http://www.mutationtaster.org/), M-CAP (http://bejerano.stanford.edu/mcap/) and CADD (https://cadd.gs.washington.edu/)^40–48^.

We confined our analysis to the primary list of genes reported to be lethal in animal models, lethal genes from earlier articles, and lists of genes incorporated in NIHF and fetal development collected by examining earlier publications and databases. In cases where no candidate variants were found, we then used Tru Sight’s gene list for inherited diseases^40^ and finally, we used all Online Mendelian Inheritance in Man (OMIM) genes (https://omim.org/downloads/).

### Confirmation of findings

All candidate variants, suspected by exome sequencing of DNA in fetuses from miscarriages, were confirmed by Sanger sequencing of the mutated point and flanking sequences in parents, using BIG Dye Terminators (Applied Biosystems 3130 Genetic Analyzer; Applied Biosystems, Foster City, CA, USA). The sequencing primers were designed using Primer3Plus (http://www.bioinformatics.nl/cgi-bin/primer3plus/primer3plus.cgi/) and Oligoanalyzer 3.1 (http://eu.idtdna.com/analyzer/applications/oligoanalyzer/). The Sanger sequencing results were analyzed by CodonCode Aligner 5.0.1 (CodonCode Corp., Dedham, MA, USA).

## Results

We collected DNA from the products of miscarriage from 20 Iranian women in consanguineous marriages and with a history of recurrent pregnancy loss. QF-PCR and a-CGH had previously been performed and reported as normal. None of the women had a specific diagnosis for pregnancy loss. As all cases were of loss of a pregnancy at less than 20 weeks of gestation, most (70%, n = 14) had no phenotype other than lethality. An autopsy was available in five cases and clinical data for these five cases are shown in Table 2. WES positively identified relevant alterations in 65% of cases (n = 13). In 45% (n = 9) of cases, we were able to classify these variants as pathogenic or likely pathogenic, while in the remaining 20% (n = 4), the candidate variants were classified as variants of unknown significance (VUS) according to the American College of Medical Genetics and Genomics guidelines (ACMG)^49^. In contrast, we were able to reach a precise diagnosis in all 5 cases (100%) with autopsies. In total, 13 rare variations including 2 de novo heterozygous, 9 inherited homozygous, and 2 inherited compound heterozygous variants were found in 11 genes; 2 genes were autosomal dominant (*COL1A1*, *SCN5A*). In silico predictions of the sequence variants are included in Supplementary Data I.

**Table 2:** Clinical characteristics of the five cases with autopsy in this study.

Case	Gender, age	Autopsy findings	Differential diagnosis	Gene
82169	Female, 18 weeks	Marked subcutaneous edema, serosal effusion including ascites and pleural effusion, cystic hygroma (non-immune hydrops fetalis)	NIHF	PIEZO1
90377	Male, 17 weeks	Small dysplastic ears, low set ears, atretic external auditory canals, hypertelorism, ankyloblepharon, absent eyebrows, small nose, small mandible, short neck, narrow thorax, distended abdomen, omphalocele, increased inter nipple distance, flexion contracture of the hips, knees and elbows, skin syndactyly of fingers, short phalanges, absent nails, syndactyly of toes (not 23), hypoplastic thumbs, hypoplastic scrotum, small penis, anal atresia, rectoperineal fistula, choanal atresia, abnormal palmar creases, severe stenosis of the trachea at the site of the vocal cords, distended trachea beyond stenosis, large hyperplastic lungs, absent right kidney, small polycystic left kidney, left hydroureter, huge megacystis, urethral atresia, bilateral club feet (varus type)	(1) Fraser syndrome; (2) Bartsocas-Papas syndrome	FRAS1
93926	Male, 18 weeks	Hypertelorism, bilateral flexion contracture of elbows and knees, bilateral clenched hands, bilateral club feet (varus type), mild rhizomelia, osteopenia, several fractures and coronal cleft, metaphyseal chondrodysplasia	(1) Metaphyseal chondrodysplasia; (2) hyperparathyroidism; (3) spondylometaphyseal dysplasia (Sedaghatian type)	COL1A1
91414	Female, 18 weeks	Low set ears, hypertelorism, adduction of left thumb, clenched hands, four ventricular hydrocephaly, atrophic cortex, hypoplastic cerebellum, severe general cortical dysplasia type II (lissencephaly type II), cerebellar cortex dysplasia, retroplacental hemorrhage	Lissencephaly type II	POMT1
97406	Female, 15 weeks	Atrophy of the limbs, body weight 91 percentile, abdominal muscle agenesis, club feet, micrognathia, hypertelorism, low set ears, mild hydrocephaly, choroid plexus cysts, muscular and corticospinal tract atrophy and kidney weight at the 99 percentile (observed to expected ratio of kidney and heart weight: 3.38, 1.99 in that order)	–	DIS3L2

In the five cases with autopsies, pathogenic/likely pathogenic variants were identified in genes *PIEZO1*, *POMT1*, *FRAS1*, *COL1A1* and *DIS3l2* (Tables 2, 3 and 4).

1. *PIEZOs* are large transmembrane ion channel proteins, and mutations in this gene have recently been reported to be a cause of hydrops fetalis^50^. *PIEZO1* has a role in urine osmolarity regulation, blood pressure control and blood vessel formation^50–54^. Mutations of this gene are associated with two diseases: (1) dehydrated hereditary stomatocytosis with/without pseudohyperkalemia and/or perinatal edema (DHS; OMIM 194380); this is an autosomal dominant disease caused by gain of function mutations; (2) hereditary lymphedema III, the generalized lymphatic dysplasia of Fotiou with non-immune fetal hydrops (GLDF; OMIM 616843), an autosomal recessive disease caused by loss of function mutations^50^. In a non-immune hydrops fetalis sample (case 82169), we detected a homozygous c.30_31delAC alteration in *PIEZO1*. This variant is a frame shift mutation in the first exon that causes nonsense-mediated decay (NMD) in translated mRNA. In this case, the biallelic loss of function of *PIEZO1* and the fetal phenotype give credence to GLDF. The parents were both carriers of this variant. According to ACMG, this variant is considered pathogenic.
2. Mutations in *FRAS1* are associated with Fraser syndrome, a rare autosomal recessive disorder. Thomas et al. (1986) proposed criteria for clinical diagnosis: major features are cryptophthalmos, syndactyly, siblings with cryptophthalmos and abnormal genitalia, and minor features include congenital malformations of the nose, ear and larynx, skeletal defects, umbilical hernia, renal agenesis and mental retardation. For diagnosis, two major or one major and four minor criteria are needed^55^. A male fetus (case 90377) with syndactyly, dysplastic ears, right kidney agenesis, club foot, flexion contracture of the hip, and atretic external auditory canals was diagnosed with a homozygous alteration in *FRAS1* (c.404C > T). The parents were confirmed to be carriers of the inherited variant. This finding was considered likely pathogenic by ACMG guidelines.
3. De novo variants in *COL1A1* gene (17q21.33) were found in two families (Families 6 and 9): p.G1169S in exon 47 (case 93926), and p.E1249G in exon 48 (case 76452). Type I collagen is encoded by the *COL1A1* and *COL1A2* genes. Collagen I is the major protein in bone, skin, and other tissue. *COL1A1* includes repeats of the Gly-X–Y triplet. Missense mutation of a conserved Gly generally leads to osteogenesis imperfecta (OI). OI is a group of skeletal dysplasia with clinical heterogeneity ranging from mild to lethal phenotype^56^. In case 93926 with mild rhizomelia, osteopenia, several fractures and a coronal cleft, and metaphyseal chondrodysplasia, a de novo mutation was detected in *COL1A1* (c.3505G > A) (p.G1169S). This mutation has been reported in the HGMD database (CM070692). Residues that substitute for glycine cause either severely debilitating or lethal phenotypes, according to Bodian et al. (2008). In this case, it may be lethal^56^.
4. A stop-gain variant found in exon 7 of *POMT1* gene (mapped to chromosome 9q34.13) in Family 7 (case 91414) was predicted to be pathogenic by ACMG guidelines. *POMT1* encodes protein O-mannosyltransferase and is associated with muscular dystrophy-dystroglycanopathy. *POMT1* and protein O-mannosyltransferase 2 (POMT2) make up the protein O-mannosyltransferase (POMT) enzyme complex^57^. Both genes are necessary to have a functioning enzyme complex^58^. This complex is abundant in fetal brain, skeletal muscles and testes^57^. Case 91414 with four ventricular hydrocephaly, atrophic cortex, hypoplastic cerebellum, severe general cortical dysplasia type II (lissencephaly type II), cerebellar cortex dysplasia and clenched hands was found to have a homozygous variation in *POMT1* (c.490C > T). This variation has been reported in the HGMD database (CM022978) and is a stop-gain resulting in NMD. Disruption of this gene in mice is lethal^59^. This variation was considered to be pathogenic.
5. Another homozygous variant in intron 3 of *DIS3L2* gene (2q37.1) in case 97406 (Family 13) was predicted to be likely pathogenic from ACMG guidelines. *DIS3L2* is associated with Perlman syndrome (#267000), an autosomal recessive disorder. Perlman syndrome is a congenital overgrowth syndrome^60^ characterized by distinctive facies, visceromegaly, abdominal wall hypoplasia, bilateral renal hamartomas, nephroblastomatosis, Wilms tumor and neonatal lethality^60,61^. A 15-week fetus (case 97406) with limb atrophy, body weight at the 91 percentile, abdominal muscle agenesis, mild hydrocephaly, choroid plexus cysts, muscular and corticospinal tract atrophy , and kidney weight at the 99 percentile (observed to expected ratio of kidney weight to heart weight: 3.38, 1.99 in that order) was found to have a homozygous alteration in *DIS3L2* (c.211-1G > A) which is predicted to disrupt the highly conserved acceptor splice site of axon 4. This alteration was classified as likely pathogenic according to ACMG guidelines.

**Table 3:** Details of variations identified in the present study.

	ID	Gene	Variant	Zygosity	Mutation effect	Origin	ACMG	Justification
1	82169	PIEZO1	PIEZO1:NM_001142864:exon1:c.30_31del:p.L10fs	Homo	Frame shift (CADD: 29, SIFT: NA, PolyPhen: NA, MutationTaster: disease causing) M-CAP: NA	Inherited	Pathogenic PSV1 PS3 PM3 PM4 PP3 PP4	Shamseldin et al.^73^ PMID: 28749478 Ranade et al.^54^ PMID: 24958852
2	84904	BBS12	BBS12:NM_152618:exon2:c.G2014A:p.A672T	Homo	Missense (CADD: 33, SIFT: deleterious, PolyPhen: damaging, MutationTaster: disease causing) align GVGD:C55 M-CAP: possibly pathogenic		VUS PM1 PM3 BS1	Hildebrandt et al. (2011) PMID: 21506742 Bardet-Biedl 12 OMIM #615989
3	90202	FUCA1	FUCA1:NM_000147:exon2:c.C404T:p.T135M	Homo	Missense (CADD: 31, SIFT: damaging, PolyPhen: damaging, MutationTaster: disease causing) align GVGD:C65 M-CAP: possibly pathogenic	Inherited	Likely pathogenic PM1 PM2 PM3 PP3	This study
4	90377	FRAS1	FRAS1:NM_025074:exon57:c.C8537A:p.A2846D	Homo	Missense (CADD: 31, SIFT: damaging, PolyPhen: damaging, MutationTaster: disease causing) M-CAP: possibly pathogenic	Inherited	Likely pathogenic PM1 PM2 PM3 PP3 PP4 BP1	Fraser syndrome OMIM #219000 Boyd et al. (1988) PMID: 2851937
5	90759	GBE1	GBE1:NM_000158:exon4:c.G467A:p.R156H GBE1: NM_000158:c.-35_-54del	Compound hetero	Missense (CADD: 35, SIFT: damaging, PolyPhen: damaging, MutationTaster: disease causing) Align GVGD:C25 M-CAP: possibly pathogenic Frameshift (CADD: NA, SIFT: NA, PolyPhen: NA, MutationTaster: disease causing)	Inherited	Likely pathogenic PM1 PM2 PM3 Pathogenic PSV1 PM2 PM3	Bruno et al. (2004) PMID: 15452297
6	76452	COL1A1	COL1A1:NM_000088:exon48:c.A3746G:p.E1249G	Hetero	Missense (CADD: 23.8, SIFT: damaging, PolyPhen: damaging, MutationTaster: disease causing) align GVGD:C65 M-CAP: possibly pathogenic	De novo	Likely pathogenic PM1 PM2 PM6 PP3	MGI: causes variable phenotype, from embryonic lethal to viable/fertile with altered fibrillogenesis
7	91414	POMT1	POMT1:NM_001136114:exon7:c.C490T:p.Q164X	Homo	Stop gain (CADD: 41, SIFT: damaging, PolyPhen: damaging, MutationTaster: disease causing) M-CAP: NA	Inherited	Pathogenic PVS1 PM2 PM3 PP4 PP5	MGI: homozygous mutation of this gene with one allele results in embryonic lethality
8	93272	STIL	STIL:NM_001048166:exon9:c.C1012T:p.H338Y	Homo	Missense (CADD: 28.1, SIFT: damaging, PolyPhen: damaging, MutationTaster: disease causing) align GVGD:C65 M-CAP: possibly benign	Inherited	VUS PM2 PP3 PM3	Izraeli et al. (1999) PMID: 10385121
9	93926	COL1A1	COL1A1:NM_000088:exon47:c.G3505A:p.G1169S	Hetero	Missense (CADD: 26.4, SIFT: damaging, PolyPhen: damaging, MutationTaster: disease causing) align GVGD:C55 M-CAP: possibly pathogenic	De novo	Likely pathogenic PS2 PM2 PP3 PP5	MGI: causes variable phenotypes, from embryonic lethal to viable/fertile with altered fibrillogenesis
10	94162	COG6	COG6:NM_020751:exon19:c.T1884G:p.Y628X	Homo	Stop gain (CADD: 38, SIFT: NA, PolyPhen: NA, MutationTaster: disease causing) M-CAP: NA	Inherited	VUS PM2 PM3	Congenital disorder of glycosylation, type IIL, OMIM #614576
11	95136	PIEZO1	PIEZO1:NM_001142864:exon45:c.C6584T:p.S2195L PIEZO1:NM_001142864:exon20:c.G2764T:p.G922W	Compound hetero	Missense (CADD: 32, SIFT: damaging, PolyPhen: damaging, MutationTaster: disease causing) align GVGD:C65 M-CAP: NA Missense (CADD: 26.7, SIFT: damaging, PolyPhen: damaging, MutationTaster: disease causing) align GVGD:C65 M-CAP: possibly pathogenic		VUS PM1 PM2 VUS PM2	Shamseldin et al.^73^ PMID: 28749478 Ranade et al.^54^ PMID: 24958852
12	94947	SCN5A	SCN5A:NM_001160161:exon21:c.C3749T:p.T1250M	Homo	Missense (CADD: 26.6, SIFT: damaging, PolyPhen: damaging, MutationTaster: disease causing) M-CAP: possibly pathogenic	Inherited	Pathogenic PS3 PM1 PM2 PM3 PP3 PP5 BP1	(HGMD CM992663) Disruption of the mouse *SCN5A* gene causes intrauterine lethality in homozygotes
13	97406	DIS3L2	DIS3L3:NM_152383.4:intron3:c.211-1G > A	Homo	Substitution (CADD: NA, SIFT: NA, MutationTaster: disease causing) M-CAP: NA	Inherited	Likely pathogenic PVS1, PM2, PP3	Perlman syndrome OMIM #267000

*ACMG* American College of Medical Genetics and Genomics, *MGI* Mouse Genome Informatics, *NA* not available, *VUS* variant of uncertain significance.

**Table 4:** Variants found in fetuses with autopsy that are likely pathogenic or pathogenic according to ACMG.

ID	Gene		Associated disease/OMIM		Gene function/disease phenotype		Phenotype
82169	PIEZO1		1-Dehydrated hereditary stomatocytosis with/without pseudohyperkalemia and/or perinatal edema (OMIM 194380)AD 2-Hereditary lymphedema III OMIM 616843 AR		*PIEZOs* are large transmembrane ion channel proteins, and mutations in this gene have recently been reported to be a cause of hydrops fetalis		Non-immune hydrops fetalis
90377	FRAS1		Fraser syndrome AR		Criteria for clinical diagnosis: major features are cryptophthalmos, syndactyly, siblings with cryptophthalmos and abnormal genitalia, and minor features include congenital malformations of the nose, ear and larynx, skeletal defects, umbilical hernia, renal agenesis and mental retardation		Small dysplastic ears, low set ears, atretic external auditory canals, hypertelorism, ankyloblepharon, absent eyebrows, small nose, omphalocele, increased inter nipple distance, flexion contracture of the hips, knees and elbows, skin syndactyly of fingers, short phalanges, absent nails, syndactyly of toes (not 23), hypoplastic thumbs, hypoplastic scrotum, small penis, anal atresia
93926	COL1A1		Osteogenesis imperfecta		Type I collagen is encoded by the *COL1A1* and *COL1A2* genes. Collagen I is the major protein in bone, skin, and other tissue OI is a group of skeletal dysplasia with clinical heterogeneity ranging from mild to lethal phenotype		Hypertelorism, bilateral flexion contracture of elbows and knees, bilateral clenched hands, bilateral club feet (varus type), mild rhizomelia, osteopenia, several fractures and coronal cleft, metaphyseal chondrodysplasia
91414	POMT1		Muscular dystrophy-dystroglycanopathy		*POMT1* and POMT2 make up the protein O-mannosyltransferase POMT enzyme complex. Both genes are necessary to have a functioning enzyme complex. This complex is abundant in fetal brain, skeletal muscles and testes		Low set ears, hypertelorism, adduction of left thumb, clenched hands, four ventricular hydrocephaly, atrophic cortex, hypoplastic cerebellum, severe general cortical dysplasia type II (lissencephaly type II), cerebellar cortex dysplasia, retroplacental hemorrhage
97406	DIS3L2		Perlman syndrome (#267000) AR		Congenital overgrowth syndrome characterized by distinctive facies, visceromegaly, abdominal wall hypoplasia, bilateral renal hamartomas and neonatal lethality		Atrophy of the limbs, body weight 91 percentile, abdominal muscle agenesis, club feet, micrognathia, hypertelorism, low set ears, mild hydrocephaly, choroid plexus cysts, muscular and corticospinal tract atrophy and kidney weight at the 99 percentile

*AD* autosomal dominant, *AR* autosomal recessive, *POMT* protein O-mannosyltransferase.

In the other 15 cases, we have no phenotype other than lethality, and possible justifying variants were found in eight cases. (Table 3) In four cases, we could classify the variant as pathogenic/likely pathogenic. These variants were found in genes *SCN5A*, *FUCA1*, *GBE1* and *COL1A1* (Table 5).

1. *SCN5A* encodes a membrane protein, which is an α-subunit of the predominant cardiac sodium channel isoform. This protein is responsible for the initial upstroke of the action potential in the heart. In case 94947, a homozygous alteration was detected in *SCN5A* (c.3749C > T). The parents were both carriers with a history of cardiac events in the family. This variant has been reported to cause long QT syndrome 3 (LQT3) (**#**603830) in the heterozygous state^62,63^.
  Homozygous mutations in *SCN5A* in mice cause intrauterine lethality mostly during organogenesis due to heart defects^64^.
2. *FUCA1* expresses a lysosomal enzyme. Fucosidosis (#230000), an autosomal recessive disorder, is a lysosomal storage disease caused by homozygous or compound heterozygous mutations in *FUCA1*. Cardinal features are coarse facies, neurological signs, visceromegaly, intellectual disability and dysostosis multiplex. There are two types of fucosidosis: type 1 is more severe and signs are seen around 6 months of age with a lifespan of a decade; type 2 is milder with longer survival^65^. In case 90202, a homozygous mutation in *FUCA1* was detected (c.404C > T). Both parents were carriers of this variant which was considered likely pathogenic.
3. *GBE1* encodes the glycogen branching enzyme. Mutations in this gene are associated with Glycogen storage disease IV (GSD IV) (#232500), an autosomal recessive metabolic disorder. GSD IV is a heterogeneous disease, which is known to have hepatic and neuromuscular features. The prenatal manifestations are fetal hydrops, polyhydramnios and decreased fetal movement^66^. Case 90759, a 13-week fetus with hydrops fetalis observed in ultrasonography, was found to have two heterozygous variants in *GBE1* gene (trans): c.467G > A and c.-35_-54del GCTCAGGCCCCACTCGACCC.
4. Case 76452 was the second case with a mutation in *COL1A1* (c.3746A > G). Mutations in this gene are associated with several OMIM diseases: Caffey disease, OMIM #114000, autosomal dominant (AD); Ehlers–Danlos syndrome, arthrochalasia type, 1, OMIM #130060, AD; Osteogenesis imperfecta, type I, OMIM #166200, AD; Osteogenesis imperfecta, type II, OMIM #166210, AD; Osteogenesis imperfecta, type III, OMIM #259420, AD; Osteogenesis imperfecta, type IV, OMIM #166220, AD. This mutation is in exon 68 and does not affect the triplet repeats and Glu has been replaced by Gly at the amino acid level. It was classified as likely pathogenic.

**Table 5:** Variants found with no autopsy that are likely pathogenic or pathogenic according to ACMG.

ID	Gene	Associated disease	Gene function/ disease phenotype
94947	SCN5A	Long QT syndrome 3 (#603830) AD Sick sinus syndrome 1(#608567) AR	Encodes a membrane protein, which is an α-subunit of the predominant cardiac sodium channel isoform. This protein is responsible for the initial upstroke of the action potential in the heart Homozygous mutations in *SCN5A* in mice cause intrauterine lethality mostly during organogenesis due to heart defects
90202	*FUCA1*	Fucosidosis (#230000) AR	lysosomal storage disease Cardinal features are coarse facies, neurological signs, visceromegaly, intellectual disability and dysostosis multiplex
90759	*GBE1*	Glycogen storage disease IV (GSD IV) (#232500) AR	GSD IV is a heterogeneous disease, which is known to have hepatic and neuromuscular features. The prenatal manifestations are fetal hydrops, polyhydramnios and decreased fetal movement
76452	*COL1A1*	Caffey disease, OMIM #114000, AD Ehlers–Danlos syndrome, arthrochalasia type, 1, OMIM #130060, AD; Osteogenesis imperfecta, type I,II,III and IV OMIM #166200, #166210, #259420, #166220, AD	

*AD* autosomal dominant, *AR *autosomal recessive.

In four cases, we found variations that are classified as variants of uncertain significance (VUS) because of the lack of phenotypic information. (Table 6) These variations were present in genes *BBS12*, *STIL*, *COG6* and *PIEZO1.*

1. A mutation in *BBS12* causes Bardet-Biedl syndrome 12 (OMIM #615989). Bardet-Biedl is a heterogeneous ciliopathy disorder with an autosomal recessive pattern of inheritance. The *BBS12* mutation is more common in the Iranian population than reported in other populations^67^. Case 84904 was found to have a homozygous mutation in *BBS12* gene (c.2014G > A). Bardet-Biedl 12 is associated with obesity and mental impairment that cannot be detected in fetal stages. This alteration was considered to be of uncertain clinical significance.
2. *STIL* is expressed in proliferating cells during early embryonic development and is necessary for mitotic spindle organization in the human cell cycle^68–71^. Alteration of this gene is associated with Primary microcephaly (#612703), an autosomal recessive disorder^71^. Izraeli et al. disrupted *STIL* in mice and the homozygous mutant caused death with neural tube defects, holoprosencephaly and left–right development abnormalities during embryonic development^70^. In case 93272, a homozygous mutation was present in *STIL* (c.1012C > T) and this was considered to be of uncertain clinical significance.
3. Mutations in *COG6* are associated with two diseases: congenital disorder of glycosylation III (**#**614576) and Shaheen syndrome (**#**615328). Shaheen syndrome is an autosomal recessive disorder characterized by intellectual disability and microcephaly. Congenital disorder of glycosylation III (CDG2L) is also autosomal recessive, characterized by intrauterine growth retardation, gastrointestinal abnormalities, infection and hematologic abnormalities. CDG2L usually results in death in infancy^72^. In case 94162, a homozygous mutation with uncertain clinical significance was detected in *COG6* (c.1884 T > G).
4. Case 95136 was found to have two mutations in *PIEZO1* (c.C6584T and c.G2764T). As discussed earlier, mutations in this gene are associated with GLDF and DHS. In this case, we have no phenotype to correlate it with our findings and thus it was considered to be of uncertain clinical significance.

**Table 6:** Variants that are VUS.

ID	Gene	Associated disease/OMIM	Gene function/disease description
84904	*BBS12*	Bardet-Biedl syndrome 12 (OMIM #615989) AR	Associated with obesity and mental impairment that cannot be detected in fetal stages
93272	*STIL*	Primary microcephaly (#612703) AR	Expressed in proliferating cells during early embryonic development and is necessary for mitotic spindle organization in the human cell cycle
94162	*COG6*	Congenital disorder of glycosylation III (**#**614576) AR Shaheen syndrome (**#**615328) AR	Congenital disorder of glycosylation III (CDG2L) is also autosomal recessive, characterized by intrauterine growth retardation, gastrointestinal abnormalities, infection and hematologic abnormalities. CDG2L usually results in death in infancy
95136	*PIEZO1*	1-Dehydrated hereditary stomatocytosis with/without pseudohyperkalemia and/or perinatal edema (DHS; OMIM 194380) AD 2-Hereditary lymphedema III, the generalized lymphatic dysplasia of Fotiou with non-immune fetal hydrops (GLDF; OMIM 616843) AR	

*AR* autosomal recessive, *AD* autosomal dominant.

## Discussion

Whole exome sequencing can detect the underlying genetic causes of disease in 25–40% of patients (pediatric and adult) with clinical indications such as congenital anomalies or developmental delay^74^. Before WES, array CGH and targeted genetic testing were performed in sporadic cases. The Middle East has the highest rate of consanguineous marriage globally due to socio-cultural factors. The prevalence of consanguineous marriage in Iran is about 38.6%^75^. Consanguineous marriage is preferred in Arab countries, particularly first cousin marriages with a rate of 20–50%^76^.

Since 2012, the possible benefit of WES for detection of the cause of miscarriage has been discussed and the results so far are promising^29^. A number of studies have been published with sample sizes of over 7 fetuses (7 to 84 cases) and these have reported a 10% to 54.5% detection rate of variants using WES in prenatal and fetal samples. In these studies, 30% to 70% of detected variants were autosomal recessive^15,74,77–83^. In our study, 84% of variants were autosomal recessive. These studies have variable inclusion criteria such as (1) single versus multiple anomalies or specific organ anomalies, or (2) singleton WES vs. trio WES. The highest diagnostic yield and pathogenic variants reported are in a study where fetuses with specific organ anomalies were studied^83^. In other studies, it was reported that trio WES increases the detection rate^84,85^. The advantages of trio WES have been reported in postnatal and prenatal studies. Trio WES allows reviewing variants with a higher probability of being pathogenic and detecting de novo variants^83^. In these studies, all fetuses had anomaly/anomalies and most cases were from unrelated couples.

To the best of our knowledge, our study is the first to employ WES on the products of miscarriage in consanguineous families with recurrent miscarriages regardless of fetal anomalies. The main difference between our study and others is in the inclusion criteria. We chose consanguineous families with spontaneous recurrent pregnancy loss (RPL) and studied the products of loss of a pregnancy at less than 20 weeks of gestation. In most cases, we had no phenotype other than lethality, and in 65% of cases, we found a possible variant. In only 45% of cases could we classify the variant as pathogenic/likely pathogenic according to ACMG guidelines. Since all fetuses were under 20 weeks gestation and some were under 10 weeks, there was no phenotype, and this made it more challenging to interpret the findings. Although all couples had RPL, we only had one product of miscarriage per family so segregation analysis was not possible due to lack of DNA samples from the other affected cases in each family. What makes interpretation of WES findings on the products of miscarriage more challenging is that Mendelian disorders in severe cases can be present as embryonic lethality, out of which many have no prenatal phenotype so mutations may not be suspected in fetuses^85^ (80).

WES on the products of miscarriage is helpful to verify lethal genes, and genes essential for embryonic development, and it expands our knowledge of prenatal phenotypes of many Mendelian disorders.

This study and others of this kind show that WES can assist in the diagnosis of the cause of miscarriage. Positive results and having a diagnosis can be useful in preconception genetic counselling for future successful pregnancies. Preimplantation genetic testing may be possible and the results may provide families with closure. After diagnosis, it is important to advise families on the risks of recurrence and their options for future pregnancies^78,86^. Identification of the cause of miscarriage will determine the risk for future pregnancies, and enable prenatal diagnosis or preimplantation genetic diagnosis for the given family. In addition, we will be able to identify lethal genes and their role in pregnancy loss. This and other studies of this kind will provide information that can assist in the assessment of repeated pregnancy loss.

## Conclusion

WES can be helpful in making a diagnosis of lethal disorders (especially autosomal recessive disorders) in consanguineous couples after prior genetic testing (QF-PCR and a-CGH).

## Supplementary Information

Supplementary Dataset.
